# Caffeic acid phenethyl amide improves glucose homeostasis and attenuates the progression of vascular dysfunction in Streptozotocin-induced diabetic rats

**DOI:** 10.1186/1475-2840-12-99

**Published:** 2013-07-06

**Authors:** Yi-Jin Ho, Wen-Pin Chen, Tzong-Cherng Chi, Ching-Chia Chang Chien, An-Sheng Lee, Hsi-Lin Chiu, Yueh-Hsiung Kuo, Ming-Jai Su

**Affiliations:** 1 Institute of Pharmacology, College of Medicine, National Taiwan University, Taipei, Taiwan; 2 Graduate Institute of Medical Sciences, Chang Jung Christian University, Tainan, Taiwan; 3 Department of Medicine, Mackay Medical College, New Taipei, Taiwan; 4 Tsuzuki Institute for Traditional Medicine, China Medical University, Taichung, Taiwan

**Keywords:** Diabetes, Vascular dysfunction, Caffeic acid phenethyl amide

## Abstract

**Background:**

Glucose intolerance and cardiovascular complications are major symptoms in patients with diabetes. Many therapies have proven beneficial in treating diabetes in animals by protecting the cardiovascular system and increasing glucose utilization. In this study, we evaluated the effects of caffeic acid phenethyl amide (CAPA) on glucose homeostasis and vascular function in streptozotocin (STZ)-induced type 1 diabetic rats.

**Methods:**

Diabetes (blood glucose levels > 350 mg/dL), was induced in Wistar rats by a single intravenous injection of 60 mg/kg STZ. Hypoglycemic effects were then assessed in normal and type 1 diabetic rats. In addition, coronary blood flow in Langendorff-perfused hearts was evaluated in the presence or absence of nitric oxide synthase (NOS) inhibitor. The thoracic aorta was used to measure vascular response to phenylephrine. Finally, the effect of chronic treatment of CAPA and insulin on coronary artery flow and vascular response to phenylephrine were analyzed in diabetic rats.

**Results:**

Oral administration of 0.1 mg/kg CAPA decreased plasma glucose in normal (32.9 ± 2.3% decrease, *P* < 0.05) and diabetic rats (11.8 ± 5.5% decrease, *P* < 0.05). In normal and diabetic rat hearts, 1–10 μM CAPA increased coronary flow rate, and this increase was abolished by 10 μM NOS inhibitor. In the thoracic aorta, the concentration/response curve of phenylephrine was right-shifted by administration of 100 μM CAPA. Coronary flow rate was reduced to 7.2 ± 0.2 mL/min at 8 weeks after STZ-induction. However, 4 weeks of treatment with CAPA (3 mg/kg, intraperitoneal, twice daily) started at 4 weeks after STZ induction increased flow rate to 11.2 ± 0.5 mL/min (*P* < 0.05). In addition, the contractile response induced by 1 μM phenylephrine increased from 6.8 ± 0.6 mN to 11.4 ± 0.4 mN (*P* < 0.05) and 14.9 ± 1.4 mN (*P* < 0.05) by insulin (1 IU/kg, intraperitoneal) or CAPA treatment, respectively.

**Conclusions:**

CAPA induced hypoglycemic activity, increased coronary blood flow and vascular response to phenylephrine in type 1 diabetic rats. The increase in coronary blood flow may result from endothelial NOS activation. However, the detailed cellular mechanisms need to be further evaluated.

## Background

Diabetes is a metabolic disease resulting from defects in insulin secretion and/or insulin action and is often associated with increased risk of coronary heart disease [1]. Therefore, treating diabetes involves more than glycemic control. Chronic complications are important in diabetes and include nephropathy, neuropathy, retinopathy, and cardiovascular disease [2], with cardiovascular disease, including vascular complications [3] and cardiovascular autonomic neuropathy [4], being the major cause of morbidity and mortality in diabetic patients [5]. In addition, cardiovascular disease is the primary cause of death in patients with either type 1 or type 2 diabetes [6,7]. Patients with type 1 diabetes also bear an increased risk of coronary heart disease, and have a higher mortality from ischemic heart disease at all ages compared to the general population [2]. Therefore, development of therapeutic agents with anti-diabetic and cardiovascular protective activity is urgently required.

To evaluate the pharmacological efficacy of anti-diabetic agents, acute or chronic diabetes has been induced in animal models, such as by chemical, surgical, and genetic manipulation [8]. Streptozotocin (STZ) is the most frequently used drug to induce diabetes and has been useful for the study of multiple aspects of the disease [9]. In the cardiovascular system, studies have shown decreased basal coronary arterial flow in STZ-induced diabetic mice [10], decreased aortic blood flow in STZ-induced diabetic rats [11], and decreased sensitivity to phenylephrine of vascular tissues in type 1 diabetic rats [12]. In addition, nitric oxide (NO) production is reduced in STZ-induced diabetes, and the decrease in NO may be related to the pathogenesis of diabetic endothelial damage [13]. Normalization of NO synthase activity in type 1 diabetic rats prevents endothelial dysfunction in STZ-induced animals [14]. In long-term STZ-induced diabetic rats, the capacity of the endothelium to synthesize or release NO may decrease and disturb the sensitivity of vascular contractile response to phenylephrine [15].

Currently, effective therapeutic options exist to restore responsiveness to insulin in type 2 diabetes; however, insulin therapy is not only necessary for type 1 diabetes, but also for type 2 diabetes. Most patients with type 2 diabetes will eventually need insulin to achieve diabetes control [16]. Insulin deficiency is a common problem in both type 1 and type 2 diabetes [1]. Therefore, in our study, we used STZ-induced diabetic rats to mimic the clinical state of insulin deficiency and to evaluate new therapeutic agents.

Caffeic acid phenethyl ester (CAPE) is the major component in extracts of propolis and possesses anti-inflammatory [17], anti-viral [18], cancer cell inhibitory [19], anti-bacterial, and free radical scavenging activities [20]. CAPE can improve oxidative stress in diabetic rat hearts [21] and exert its vasorelaxation effect on aorta of rats in vitro (pEC_50_, 4.99 ± 0.19; Emax, 100.75 ± 1.65%,) [22]. Oral administration of CAPE (30 mg/kg) for 12 weeks ameliorated the atherosclerosis progress in apolipoprotein E-deficient mice [23]. In addition, intraperitoneally injected CAPE (10 μM) 1 h before reperfusion attenuated ischemia-reperfusion injury by exerting antioxidant activity in Wistar rats [24]. CAPE significantly decreased the fasting blood levels of glucose, alanine aminotransferase, cholesterol, and triglyceride induced by diabetes [25]. Several natural products exhibit powerful glucose lowering activity, such as caffeic acid [26], extracts of propolis from north China [27], extracts of propolis from Brazil [28], capsaicin [29], and curcumin [30].

Recently, a CAPE analogue, caffeic acid phenethyl amide (CAPA), with amide linkage between caffeic acid and phenethyl group that resists hydrolysis in the circulation, was found to be more stable compared to CAPE in rat plasma [31] and to possess cytoprotective effects against H_2_O_2_-induced cell death in human umbilical vascular endothelial cells [32]. In addition, CAPA has shown α-glucosidase inhibitory effects in yeast [33], adiponectin productive activity in 3T3-L1 cells [34], as well as antihyperglycemic activity and cardioprotection effects in diabetic mice [10,35]. It also attenuates cardiac dysfunction in abdominal aortic banding-induced cardiac hypertrophy [36], indicating that CAPA may be beneficial to treat diabetes and cardiovascular complications.

The aim of this study was to characterize the acute effect of CAPA on vascular function and glucose homeostasis in normal and type 1 diabetic rats. In addition, the chronic effect of CAPA on vascular dysfunction of type 1 diabetic rats was investigated and compared to that of insulin.

## Methods

### Compound

**Figure 1:**
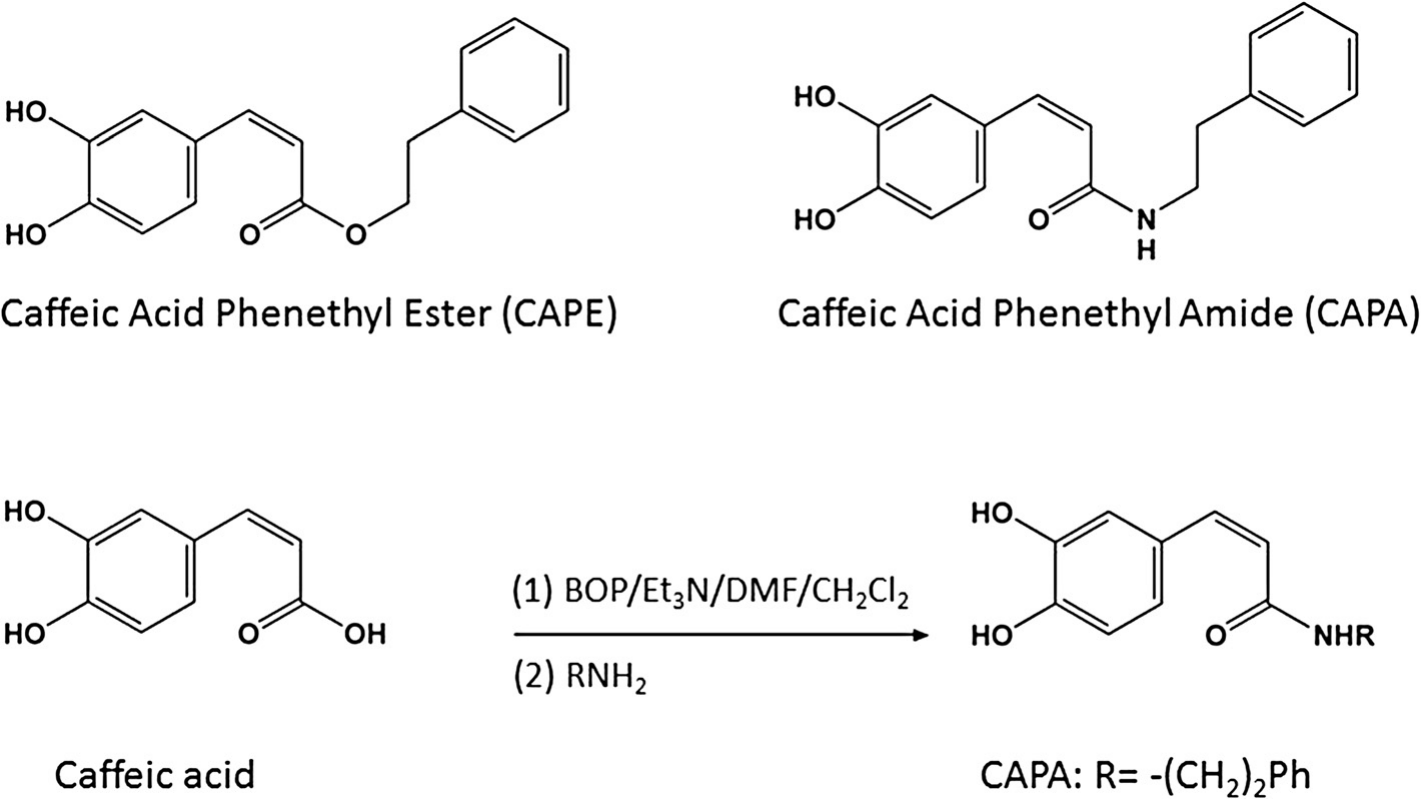
**The structures of CAPE and CAPA**, **and the synthetic process of CAPA.** CAPA was obtained from the amide binding coupling method, beginning with caffeic acid. CAPA: R=−(CH_2_)_2_Ph. benzotriazol-1-yloxytris (dimethylamino) phosphonium hexafluorophosphate (BOP), dichloromethane (CH_2_Cl_2_), triethylamine (Et3N), dimethylformamide (DMF).

To synthesize CAPA (Figure 1), a solution of benzotriazol-1-yloxytris (dimethylamino) phosphonium hexafluorophosphate (1.2 equiv) in dichloromethane (CH_2_Cl_2_) (5 mL) was added to a mixture of caffeic acid (100 mg), R-NH_2_ (1.2 equiv), and triethylamine (0.08 mL) in dimethylformamide (1.0 mL). The mixture was stirred at 0°C for 30 min and then at room temperature for 12 h. This reaction mixture was evaporated under vacuum, and the residue was partitioned between ethyl acetate (AcOEt) and H_2_O. The AcOEt layer was washed with 3 N aqueous HCl and 10% NaHCO_3_ (aq), dried over MgSO_4_, and vacuum concentrated. The residue was further purified by column chromatography using an eluting solution (CH_2_Cl_2_-AcOEt 1:1, v/v) on silica gel (70–230 and 230–400 mesh, Merck 7734). The final products (82–88% yield) were recrystallized from AcOEt to obtain pure crystals. ^1^H and ^13^C nuclear magnetic resonance (NMR) spectra were recorded on a Bruker Avance 500 spectrometer, electron impact mass spectra were determined on a Finnigan TSQ-46C mass spectrometer, and infrared spectra were recorded on a NicoletMagna-IR 550 spectrophotometer. CAPA was obtained from R substitution with -(CH_2_)_2_Ph. CAPA: solid; melting point 148–149°C. Infrared ν_max_ (cm^-1^): 3288, 1642, 1591, 1523, 1361, 1279, 1036, 975, 849. ^1^H NMR (CD_3_COCD_3_, 500 MHz): δ 2.84 (2H, t, *J* = 6.8 Hz), 3.53 (2H, q, *J* = 6.8 Hz), 6.43 (1H, d, *J* = 15.2 Hz), 6.83 (1H, d, *J* = 8.1 Hz), 6.92 (1H, dd, *J* = 8.1, 1.8 Hz), 7.07 (1H, d, *J* = 1.8 Hz), 7.15–7.30 (5H, m), 7.35 (1H, br s, -NH), 7.43 (1H, d, *J* = 15.2Hz), 8.20 (1H, s,-OH), 8.42 (1H, s,-OH). EI-MS *m*/*z* (%): 283 (M^+^, 17), 178 (22), 163 (100).

### Chemicals

STZ, pentobarbital, Nω-nitro-l-arginine methyl ester (l-NAME), methylene blue, phenylephrine, and dimethylsulfoxide (DMSO) were purchased from Sigma-Aldrich, USA. The inhibitor of NO-sensitive guanylyl cyclase, ODQ (1*H*-[1,2,4] Oxadiazolo [4,3-*a*] quinoxalin-1-one) was purchased from Tocris Bioscience, USA. The chemicals for the physiological solution were purchased from J.T. Baker, Capital Scientific Inc. and Wako Pure Chemical Industries, Japan. Insulin (Insulin Zinc Suspension, Monotard® HM 100 IU/mL) was purchased from Novo Nordisk A/S, Denmark.

### Animals

We used 8-week-old male Wistar rats weighing 250–300 g (bred in a Lab animal center, National Taiwan University, Taiwan) for evaluation of hypoglycemic activity, insulin secretion activity, glucose tolerance test, measurement of coronary arterial flow rate, aortic contractile response, and induction of diabetes. All animal procedures were performed according to the *Guide for the Care and Use of Laboratory Animals* of the National Institutes of Health, as well as the guidelines of the Animal Welfare Act, and the animal studies were approved with a certificate number 20110073 by the Institutional Animal Care and Use Committee of the College of Medicine, National Taiwan University. For induction of diabetes, rats were anesthetized with sodium pentobarbital (30 mg/mL), after a 72-h fast [37] and administered STZ (freshly dissolved in sterile, non-pyrogenic 0.9% NaCl solution in a volume of 1 mL/kg body weight) intravenously through the tail vein at a single dose (60 mg/kg) [38]. Two weeks after STZ injection, animals were considered to have type 1 diabetes if they had plasma glucose levels higher than 350 mg/dL and other diabetic features, such as polyuria, polydipsia, and hyperphagia [39].

### Effect of CAPA on plasma glucose in normal and STZ-induced diabetic rats

We administered CAPA (suspended in distilled water in a volume of 1 mL/kg body weight) orally by gavage to overnight-fasted rats at different doses of 0.1 mg/dL, 0.5 mg/dL, and 1 mg/dL (*n* = 4 to 11). In a previous study, rats that received sodium pentobarbital showed no changes in plasma glucose [40]. Thus, under anesthesia with sodium pentobarbital (30 mg/kg intraperitoneal), blood samples (0.2 mL) were collected from the femoral vein to measure plasma glucose levels. The blood samples were centrifuged at 1000 *g* for 5 min, and 10 μL of clear supernatant serum was added from the 1 mL glucose kit (Biosystems S.A., Barcelona, Spain). We then estimated the levels of plasma glucose by a spectrophotometer (BTS-330, Biosystems S.A., Barcelona, Spain), run in duplicate [41]. The time course of the effect of CAPA on plasma glucose in STZ-induced diabetic rats was preliminarily determined; the plasma glucose-lowering effect of CAPA at an oral dosage of 0.5 mg/kg reached a plateau within 90 min and was maintained until 120 min. Thus, we measured the plasma glucose decreasing effects of CAPA using blood samples collected 90 min after oral administration. For the control group, animals were orally administered the same volume of distilled water used in CAPA suspension.

### Effects of CAPA on insulin secretion

We measured plasma insulin levels using an insulin enzyme linked immunosorbent assay (ELISA) kit (Rat Insulin ELISA; Mercodia AB, Uppsala, Sweden) [42]. Briefly, 8-week-old Wistar rats (250–300 g body weight, *n* = 8) were anesthetized with sodium pentobarbital (30 mg/kg intraperitoneal), and blood samples (0.2 mL) were collected from the femoral vein, centrifuged at 1000 *g* for 5 min and 10 μL of clear supernatant serum was used to measure plasma insulin levels. After completing the ELISA test procedure, we estimated the levels of insulin using a spectrophotometer (Victor^3^, PerkinElmer Inc., MA, USA); samples were run in duplicate.

### Effect of CAPA on intravenous glucose tolerance test

For the intravenous glucose tolerance test, overnight fasted rats were anesthetized with sodium pentobarbital (30 mg/kg intraperitoneal). We administered CAPA (0.5 mg/mL/kg) in a volume of 1 mL/kg body weight (*n* = 6) or the same volume of distilled water (vehicle treatment, *n* = 8) 30 minutes before intravenous injection of glucose (1 g/kg body weight) and measured the blood glucose level at 5, 10, 20, 40, 60, 90, and 120 min after glucose injection [43].

### Effect of CAPA on coronary arterial flow rate in Langendorff-perfused hearts

The rats were anesthetized with sodium pentobarbital (50 mg/kg) and given heparin (300 IU/kg) intraperitoneally. The Langendorff-perfused heart model, using a constant perfusion pressure instead of constant flow rate, was employed [44]. Hearts were rapidly excised and immersed in perfusion medium. After excision of the heart from the chest, the aorta was cannulated and perfused at 80 mmHg with perfusate containing 119.7 mM NaCl, 23.8 mM NaHCO_3_, 5.0 mM KCl, 0.3 mM NaH_2_PO_4_, 1.2 mM CaCl_2_, 1.1 mM MgCl_2_, and 5.6 mM glucose. The perfusate was equilibrated with 95% O_2_, 5% CO_2_ at 37°C. Perfusion pressure and flow rate (mL/min) were monitored by a MLT844/D pressure transducer (Capto, Horten, Norway), and electrocardiogram was digitally measured during the period of the experiment by a data acquisition device (PowerLab, ADInstruments, Castle Hill, Australia). The 2 tips of the ventricular recording electrode were separated and placed on opposite sides of the ventricular epicardium to generate a bipolar transcardiac electrogram. RR intervals were measured as the average of 6 consecutive cycles to calculate the heart rate as beats per minute [45].

### Effect of CAPA on thoracic aorta

We used the thoracic aorta of rats for vascular contraction response studies. The thoracic aorta were cleaned of adhering periadventitial fat, cut into 3-mm length rings, and then incubated in an organ bath containing Kreb’s buffer (composition [in mM]: NaCl 118.2, KCl 4.7, KH_2_PO_4_ 1.2, NaHCO_3_ 25, MgSO_4_ 1.2, CaCl_2_ 1.9, and glucose 11.7), with a pH of 7.4, and gassed with 95% O_2_ and 5% CO_2_. The aortic rings were mounted to a polygraph (model RS 3400 recorder, Gould), and contraction force was monitored by a pressure transducer (Type BG 25; Gould, Oxnard, Calif., USA). Aortic rings were equilibrated at a resting tension of 2 g for 1 h before the experiments [46]. We evaluated the relaxation effect of CAPA on endothelium-intact and endothelium-denuded aorta, pre-constricted with phenylephrine 1 μM or KCl 80 mM. Contractile responses were generated by addition of incremental concentrations of phenylephrine (0.001 μM to 10 μM) on endothelium-denuded aortic rings. The intactness or absence of endothelium was confirmed by the relaxant responses to acetylcholine (1 μM) in rings precontracted with phenylephrine 1 μM [47].

### Vascular effects of CAPA chronic treatment on STZ-induced diabetic rats

A previous study demonstrated that STZ-induced diabetic mice expressed early vascular dysfunction [48], increased blood glucose stability, and decreased E/A flow ratio [49] after 4 weeks of STZ-induction. We therefore started the therapeutic regimen from this time point (4 weeks). There were 3 treatment groups of diabetic rats: vehicle-treated group (0.1 mL/kg DMSO), insulin-treated group (1 IU/kg insulin), and CAPA-treated group (3 mg/kg CAPA). All groups were administered the agents intraperitoneally twice daily for 4 weeks. The control group consisted of age- and sex-matched normal Wistar rats.

### Data analysis

Values are expressed as means ± SEM of *n* observations, where *n* represents the number of animals studied. The data were subjected to ANOVA followed by a multiple-comparison test (Bonferroni’s test) or analyzed by unpaired Student’s *t*-test. *P* < 0.05 was considered to be statistically significant.

## Results

### CAPA decreased plasma glucose levels in normal and STZ-induced diabetic rats

The effect of CAPA on plasma glucose levels was measured in anesthetized overnight-fasted rats. The maximum plasma glucose lowering effect was observed at 90 minutes after oral administration of 0.1 mg/kg CAPA (*n* = 7) compared to vehicle treatment (*n* = 8) (percent decrease in glucose level, 32.9 ± 2.3% vs. 5.8 ± 5.7%, *P* < 0.05) (Table 1). The plasma glucose lowering effect of CAPA in normal rats was not dose dependent (32.8 ± 3.4%, *n* = 6 and 29.5 ± 1.7%, *n* = 6 after oral administration of 0.5 mg/kg and 1 mg/kg CAPA, respectively). Obvious plasma glucose lowering activity was observed after oral administration of 0.1 mg/kg CAPA in STZ-induced type 1 diabetic rats (11.8 ± 5.5%, *n* = 4, *P* < 0.05 compared to vehicle treatment, 0.6 ± 0.1%, *n* = 8). No further increase in plasma glucose lowering activity was observed when the dose was increased to 0.5 mg/kg and 1.0 mg/kg (13.8 ± 4.3%, *n* = 7 and 11.6 ± 1.7%, *n* = 11, respectively).

**Table 1:** Effect of CAPA on plasma glucose levels in normal and diabetic rats

**CAPA (mg/kg)**	**Normal rats**				**Type 1 diabetic rats**			
	**Plasma glucose**		**% decrease in plasma glucose**	**n**	**Plasma glucose**		**% decrease in plasma glucose**	**n**
	**Pre-treatment (mg/dL)**	**Post-treatment (mg/dL)**			**Pre-treatment (mg/dL)**	**Post-treatment (mg/dL)**		
Vehicle	138.5 ± 5.9	129.3 ± 2.2	5.8 ± 5.7	8	446.6 ± 11.6	444.1 ± 11.6	0.6 ± 0.1	8
0.1	124.3 ± 4.6	83.1 ± 3.7 *	32.9 ± 2.3 *	7	469.8 ± 37.2	409.8 ± 37.9	11.8 ± 5.5 *	4
0.5	120.5 ± 5.3	81.0 ± 5.7 *	32.8 ± 3.4 *	6	461.7 ± 21.3	394.0 ± 13.5 *	13.8 ± 4.3 *	7
1	126.2 ± 2.0	89.0 ± 3.2 *	29.5 ± 1.7 *	6	447.4 ± 25.5	393.7 ± 20.6 *	11.6 ± 1.7 *	11

Plasma glucose was measured at 90 min after oral administration of vehicle or CAPA in overnight fasted rats. Rats treated with vehicle were used as controls. Data (mean ± SEM) were obtained from 4–11 animals. *P < 0.05 compared with the vehicle control group and examined by the paired *t*-test.

### CAPA increased insulin secretion

Insulin secretion was measured in overnight fasted and anesthetized rats. Plasma insulin concentration increased after 30 minutes from a mean value of 7.7 ± 1.0 μIU/mL to 14.9 ± 3.4 μIU/mL (*n* = 8) after oral administration of 1 mg/kg CAPA (Table 2).

**Table 2:** Effect of CAPA on plasma insulin levels in normal rats

	**Plasma insulin**		
**CAPA (mg/kg)**	**Pre-treatment**	**Post-treatment**	**n**
	**(μIU/mL)**	**(μIU/mL)**	
1	7.7 ± 1.0	14.9 ± 3.4*	8

Plasma insulin was measured at 30 min after oral administration of CAPA (1 mg/kg) in overnight fasted rats. Data (mean ± SEM) were obtained from 8 animals. *P < 0.05 compared with the pre-treatment animals and examined by the paired *t*-test.

### CAPA improved glucose tolerance

**Figure 2:**
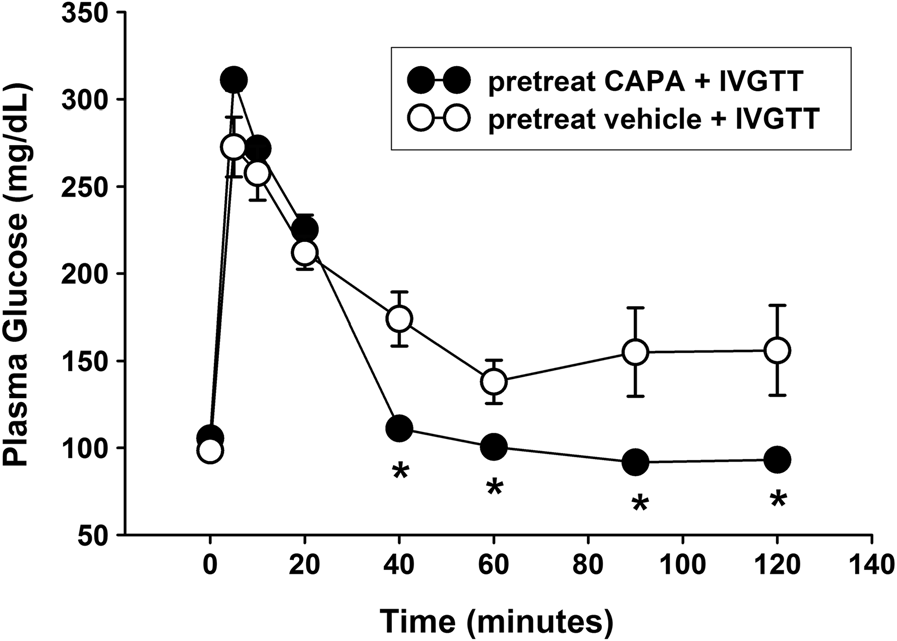
**The effect of CAPA on the glucose tolerance test.**Intravenous glucose tolerance tests (IVGTT) were performed in anesthetized fasted animals. Rats were orally administered CAPA (0.5 mg/mL/kg, *n* = 6) or the same volume of distilled water (vehicle, *n* = 8) at 30 minutes before intravenous injection of glucose (1000 mg/kg). Blood glucose levels were measured before and at 5, 10, 20, 40, 60, 90, and 120 min after glucose injection. (●) CAPA-treated group: CAPA (0.5 mg/mL/kg, p.o. 30 min before glucose intravenous injection). (○) Vehicle-treated group: Rats treated with vehicle at the same volume were used as controls. Data are presented as the mean ± SEM. *P < 0.05 compared with the vehicle.

The plasma glucose levels of CAPA (0.5 mg/kg)-treated rats (*n* = 6) were 112.2 ± 4.3, 100.5 ± 3.6, 91.7 ± 2.1, and 93.2 ± 2.7 mg/dL at 40, 60, 90, and 120 minutes after intravenous glucose (1000 mg/kg) injection, respectively; these levels were significantly (*P* < 0.05) lower than those of the distilled water treated group (174.0 ± 15.4 mg/dL, 138.0 ± 12.4 mg/dL, 155.0 ± 25.3 mg/dL, and 156.0 ± 25.7 mg/dL, respectively, *n* = 8, Figure 2).

### CAPA increased coronary arterial flow rate

**Figure 3:**
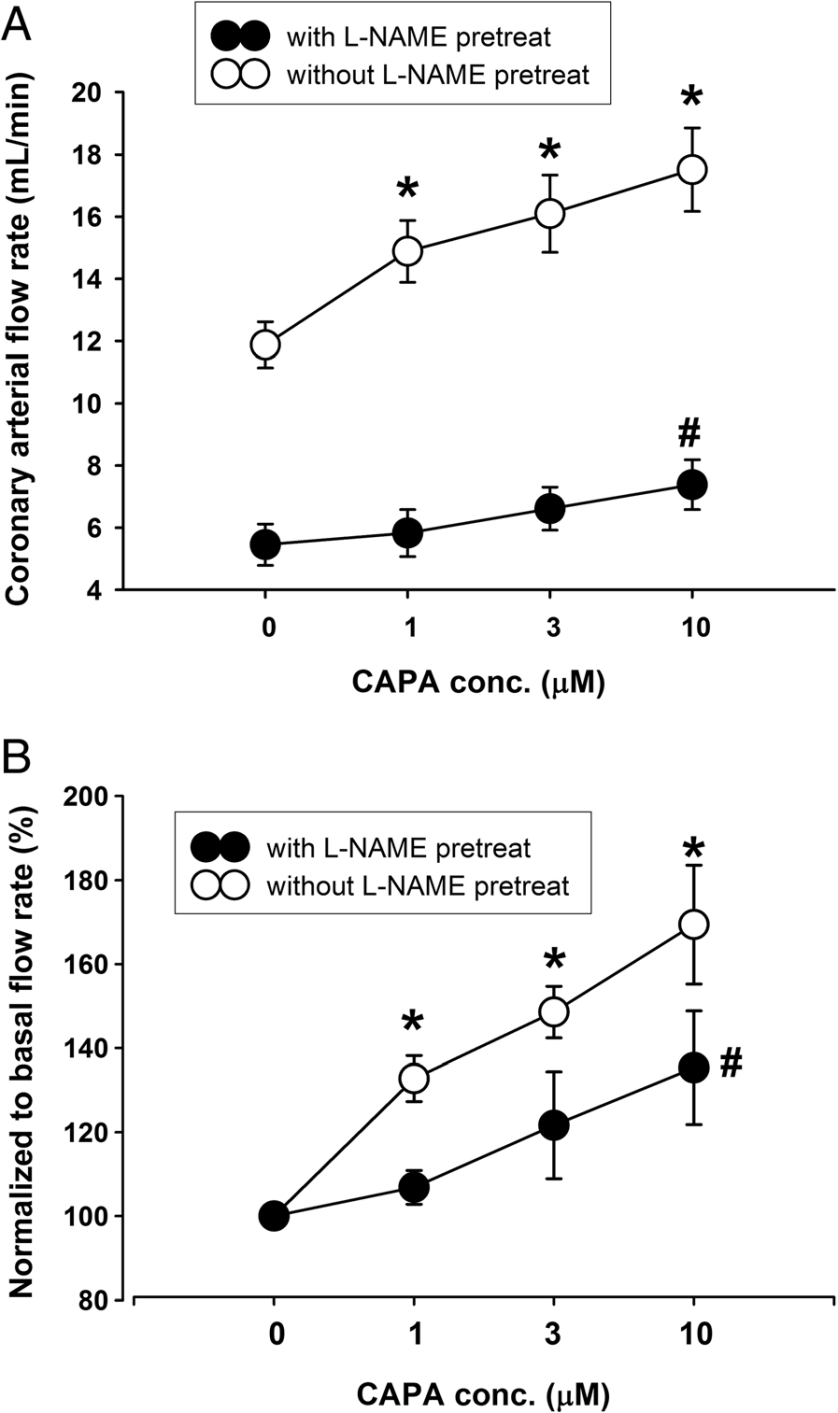
**Effect of CAPA on coronary arterial flow rate in normal or l**-**NAME**-**treated rat hearts.** Coronary arterial flow rate (mL/min) was measured after 30 min of equilibrium with retrograde perfusion using a constant pressure of 80 mmHg. The l-NAME-treated hearts were perfused with 10 μM l-NAME **(A)**, and data were normalized to basal flow rate **(B)**. Data (mean ± SEM) were obtained from 4–9 animals. *P < 0.05 compared with the untreated control of normal rat hearts and ^#^*P* < 0.05 compared with the untreated control of the L-NAME group.

Coronary arterial flow rate was measured 20 minutes after perfusion with a solution containing 1, 3, or 10 μM CAPA. The flow rate of perfused hearts compared to normal rats was shown to be increased by 1 μM CAPA from 11.8 ± 1.0 mL/min to 15.6 ± 1.2 mL/min. Furthermore, coronary arterial flow rate dose-dependently increased to 17.4 ± 1.3 mL/min and 19.5 ± 0.7 mL/min following injection of 3 and 10 μM CAPA, respectively (Table 3). CAPA 1, 3, and 10 μM also increased the coronary arterial flow rate in the hearts of STZ-induced type 1 diabetic rats from 10.3 ± 0.6 mL/min to 11.9 ± 0.9 mL/min, 13.3 ± 1.1 mL/min, and 14.8 ± 1.4 mL/min, respectively. This increase in coronary arterial flow rate, however, was less than that in normal rats. The heart rate of animals during all experiments did not significantly change (Table 3). Both the basal coronary arterial flow rate and the coronary arterial flow rate in the presence of CAPA (1, 3, or 10 μM) in normal rats were significantly reduced by l-NAME (10 μM), a nitric oxide synthase (NOS) inhibitor, from 10.8 ± 0.8 mL/min, 13.2 ± 0.8 mL/min, 14.0 ± 1.0 mL/min, and 16.8 ± 1.2 mL/min to 5.5 ± 0.7 mL/min, 5.8 ± 0.8 mL/min, 6.6 ± 0.7 mL/min, and 7.4 ± 0.8 mL/min, respectively (Figure 3A and normalized response in Figure 3B). The increase in coronary arterial flow rate by 1 and 3 μM CAPA was mostly abolished by 10 μM l-NAME.

**Table 3:** Effect of CAPA on the coronary arterial flow rate and heart rate

**CAPA (μM)**	**Normal rat hearts**			**Type 1 diabetic rat hearts**		
	**Coronary arterial flow rate (mL/min)**	**Heart rate (BPM)**	**n**	**Coronary arterial flow rate (mL/min)**	**Heart rate (BPM)**	**n**
0	11.8 ± 1.0	236.1 ± 7.1	5	10.3 ± 0.6	207.8 ± 12.9	9
1	15.6 ± 1.2 *	234.2 ± 11.7	5	11.9 ± 0.9 *	208.5 ± 11.1	9
3	17.4 ± 1.3 *	234.1 ± 6.5	5	13.3 ± 1.1 *	203.2 ± 9.2	8
10	19.5 ± 0.7 *	218.0 ± 5.5	4	14.8 ± 1.4 *	205.5 ± 10.2	6

Effect of CAPA on the coronary arterial flow rate (mL/min) and heart rate (*BPM*: beats per minute) in normal and type 1 diabetic rat hearts. Coronary arterial flow rate was measured after 30 min of equilibrium with retrograde perfusion at a constant pressure of 80 mmHg. Data (mean ± SEM) were obtained from 4–9 animals. *P < 0.05 compared with CAPA (0 μM)-administered animals.

### CAPA relaxed the thoracic aorta and shifts the dose–response curve of Phenylephrine-induced contraction

**Figure 4:**
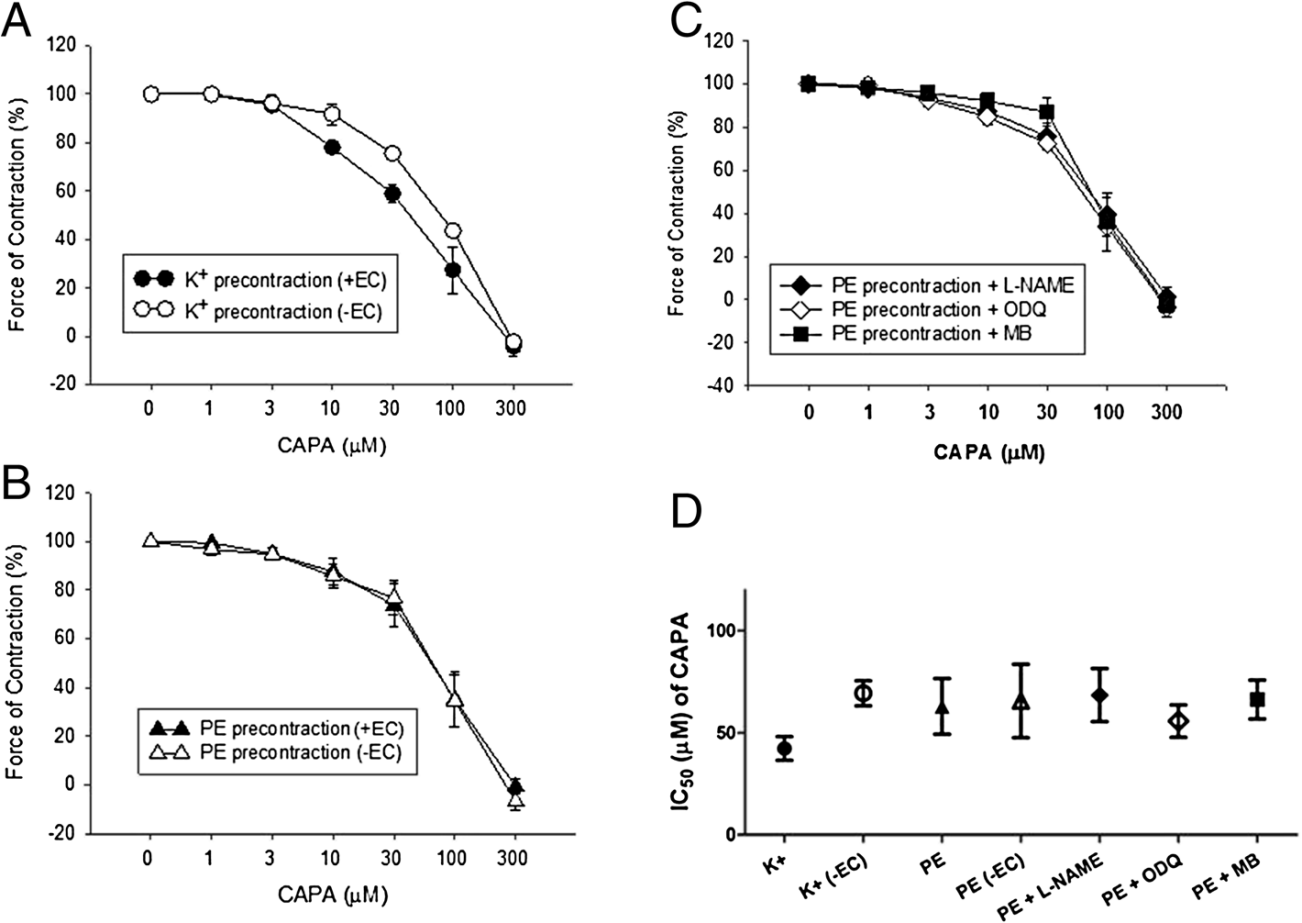
**Concentration**-**dependent inhibition of CAPA on thoracic aorta**, **constriction**-**induced by high concentration potassium or phenylephrine.** The relaxation effect of CAPA was measured in endothelium-intact (+EC) and endothelium-denuded (−EC) thoracic aorta. The aortic strips were pre-constricted with high concentration of potassium (80 mM) **(A)** or 1 μM phenylephrine **(B)**, pre-treated with inhibitors **(C)**, such as 10 μM NOS inhibitor (l-NAME), 10 μM nitric oxide-sensitive guanylyl cyclase selective inhibitor (ODQ), and 100 μM soluble guanylyl cyclase inhibitor (MB), and CAPA was added in a cumulated concentrations in the organ bath to assess relaxation effects. The IC_50_ values for CAPA are shown in **(D)**. Data (mean ± SEM) were obtained from 6–8 animals.

**Figure 5:**
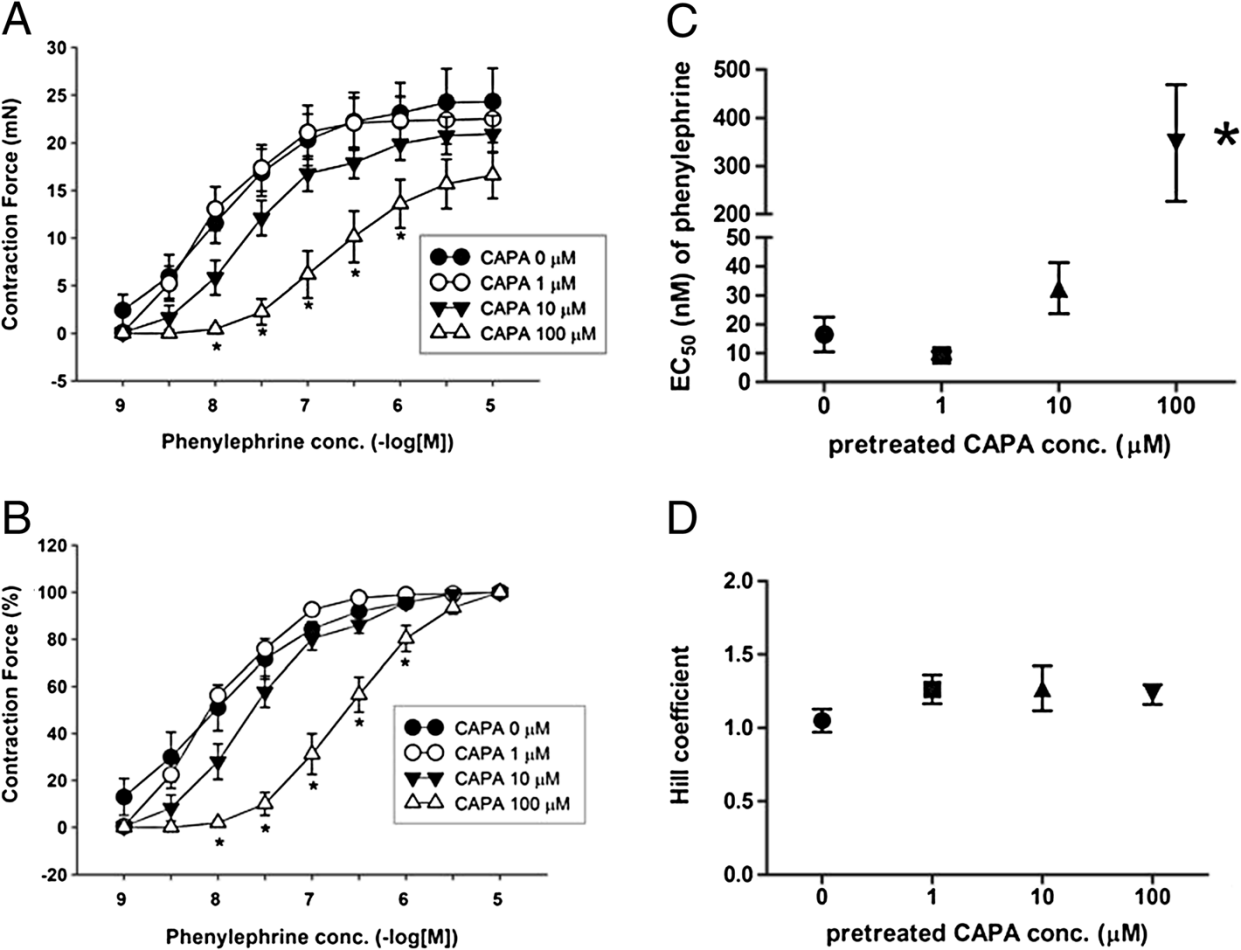
**Effect of CAPA on concentration**-**dependent contractile response to phenylephrine in endothelium**-**denuded aortic strips.** The effect of CAPA on the contractile response to phenylephrine was measured in endothelium-denuded thoracic aorta. The aortic strips were pre-treated with 1, 10 and 100 μM CAPA, and phenylephrine was added in a cumulative dose in the organ bath **(A)**. Normalized contraction to the maximum response of phenylephrine is shown **(B)**. The EC_50_ values for phenylephrine and the Hill coefficient are shown in **(C)** and **(D)**. Data (mean ± SEM) were obtained from 6–8 animals. *P < 0.05 compared with the vehicle, DMSO pre-treatment group.

CAPA showed concentration-dependent inhibition of endothelium-intact and endothelium-denuded rat thoracic aorta, constriction induced by 80 mM potassium or 1 μM phenylephrine (Figure 4A and Figure 4B). These vasorelaxant effects precontracted with phenylephrine were not affected by 10 μM NOS inhibitor (l-NAME), 10 μM NO-sensitive guanylyl cyclase selective inhibitor (ODQ), or 100 μM soluble guanylyl cyclase inhibitor methylene blue (Figure 4C). The IC_50_ values for inhibition of vasoconstriction are shown in Figure 4D, with no significant differences seen among them. The concentration-response curve of endothelium-denuded aortic strips to phenylephrine demonstrated a significant right shift (Figure 5A and normalized response in Figure 5B), and the EC_50_ of phenylephrine was significantly increased by 100 μM CAPA (EC_50_ = 347.7 ± 120.9 nM) (Figure 5C); the Hill coefficient of phenylephrine-induced contraction remained unchanged (Figure 5D).

### CAPA attenuated the progression of vascular dysfunction in STZ-induced diabetic rats

**Figure 6:**
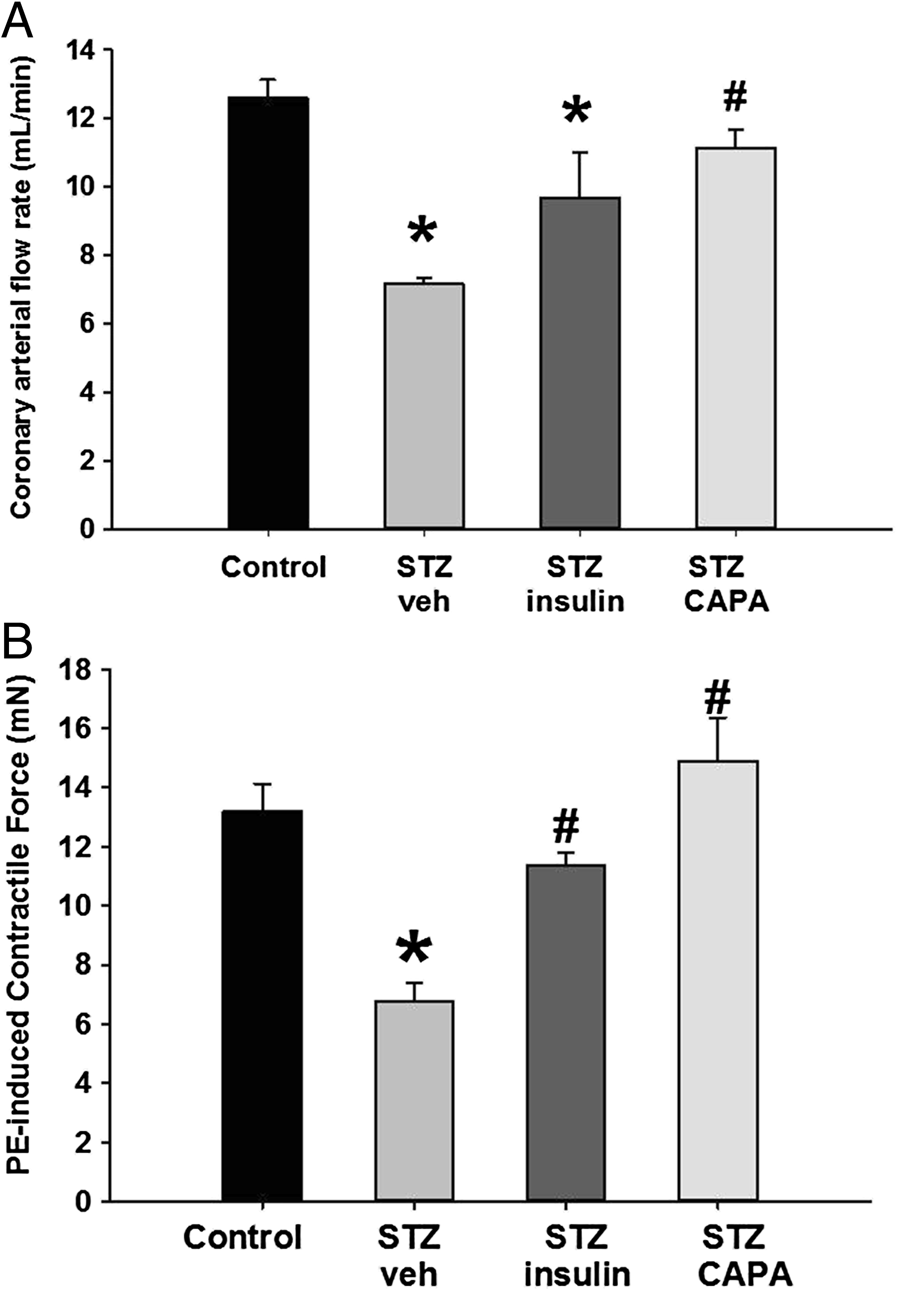
**Effect of chronic treatment with CAPA and insulin on the vascular response. (A)** The basal coronary arterial flow rate (mL/min) in retrograde-perfused hearts was measured to evaluate the effect of CAPA in type 1 diabetic rats. The animals were divided into 4 groups and intraperitoneally administered CAPA twice daily for 4 weeks. Control: age- and sex-matched normal rats, *n* = 6. STZ-veh: vehicle-treated diabetic rats, 0.1 mL/kg DMSO, *n* = 4. STZ-insulin: insulin-treated diabetic rats, 1 IU/kg, *n* = 4. STZ-CAPA: CAPA-treated diabetic rats, 3 mg/kg, *n* = 5. ^*,#^*P* < 0.05 compared with control groups and STZ-induced diabetic rats treated with vehicle. **(B)** Effect of CAPA on the phenylephrine-induced aortic constriction of type 1 diabetic rat aorta. The effect of CAPA on the phenylephrine-induced aortic contraction response (mN) of STZ-induced type 1 diabetic rat thoracic aorta was measured in isolated aortic strips. The animals were divided into 4 groups. Control: age- and sex-matched normal rats, *n* = 4. STZ-veh: vehicle-treated diabetic rats, 0.1 mL/kg DMSO, *n* = 4. STZ-Ins: insulin-treated diabetic rats, 1 IU/kg, *n* = 4. STZ-CAPA: CAPA-treated diabetic rats, 3 mg/kg, *n* = 6. ^*,#^*P* < 0.05 compared with control and STZ-veh groups.

Compared to a coronary arterial flow rate of 12.6 ± 0.5 mL/min (*n* = 6) in control normal rats, the mean flow rate in diabetic rats at 8 weeks after STZ induction was reduced to 7.2 ± 0.2 mL/min (*n* = 4, *P* < 0.05). However, twice daily treatment with CAPA (3 mg/kg, intraperitoneal) in diabetic rats (*n* = 5, started at 4 weeks after STZ induction) for 4 weeks significantly increased coronary arterial flow rate to 11.2 ± 0.5 mL/min (*P* < 0.05 compared with STZ-vehicle treatment) (Figure 6A). In addition, the contractile force induced by 1 μM phenylephrine was significantly decreased in the STZ-vehicle group (from 13.2 ± 0.9 mN to 6.8 ± 0.6 mN, *n* = 4, *P* < 0.05), but increased from 6.8 ± 0.6 mN to 11.4 ± 0.4 mN (*n* = 4) and 14.9 ± 1.4 mN (*n* = 6) by insulin or CAPA treatment, respectively (*P* < 0.05 compared with STZ-vehicle treatment) (Figure 6B).

## Discussion

CAPA was found to decrease plasma glucose levels in normal and diabetic rats. Decreased plasma glucose levels in normal rats are associated with both enhancement of insulin secretion and glucose utilization. Several pharmacological agents such as K_ATP_ channel inhibitors [50], Glucagon-like peptide-1 and dipeptidyl peptidase IV (DPP-IV) inhibitors [51], are known to exert their hypoglycemic action via enhancement of insulin release. In a previous study, we tested the effect of CAPA on DPP-IV activity and K_ATP_ potassium current in a pancreatic beta cell line, MIN6. CAPA did not inhibit DPP-IV activity, but was found to inhibit K_ATP_ channels with a calculated IC_50_ of 21.2 μM (data not shown). Since the calculated maximal plasma concentration after oral administration of CAPA at 0.5 mg/kg is less than 1.8 μM, the stimulation of insulin secretion after oral administration can only partly be attributed to its inhibition of K_ATP_ channels. In addition, in the present study, CAPA was found to inhibit α-adrenergic receptors on vascular tissue. Since inhibition of sympathetic α-adrenergic receptors is reported to enhance insulin release [52], the stimulation of insulin release by CAPA may be partly attributed to inhibition of α-adrenoceptors. In STZ-induced type 1 diabetic rats, CAPA lowered plasma glucose levels even though this diabetic rat model has low insulin secretion activity, suggesting that insulin-independent mechanisms may be involved. Our findings were concordant with those studies that reported plasma glucose lowering activity of CAPA in STZ-induced type 1 diabetic mice [35] and increased glucose transporter 4 protein expression along with insulin-induced glycogen synthesis in diet-induced type 2 diabetic mice [10]. The detailed mechanism responsible for the antidiabetic activity in type 1 diabetic rats remains to be investigated.

Diabetes is associated with several cardiovascular risk factors, such as abnormal glycemia, lipidemia, visceral obesity, and oxidative stress, which impair endothelial function and predispose patients to macrovascular disease, including coronary artery disease and cerebral vascular disease, ultimately the major causes of morbidity and mortality in diabetic patients [2]. A previous study showed that CAPA has cytoprotective effects on human umbilical vein endothelial cells [32]. In the present study, we found that 1 and 3 μM CAPA increased coronary blood flow in a Langendorff-perfused heart model. In addition, we observed that the CAPA-induced increase in coronary blood flow was prevented by an NOS inhibitor. Our results suggest that CAPA may enhance coronary blood flow by increasing the availability or level of NO.

Our results suggest that CAPA may enhance coronary flow by increasing the availability of NO in the coronary artery. The 2,2-diphenyl-1-picrylhydrazyl radical scavenging activity of CAPA, as shown by an EC_50_ of 18.6 ± 3.2 μM (comparable to the EC_50_ of 15.6 ± 2.0 μM of CAPE, data not shown), may reduce tissue oxidative stress and increase tissue availability of NO. In addition, our studies in type 2 diabetic mice show that CAPA treatment could increase manganese superoxide dismutase in fat tissue [10], but whether this effect occurs in the coronary vascular bed remains unknown.

Higher concentrations of CAPA were found to relax the thoracic aorta following potassium- or phenylephrine-induced constriction. None of the inhibitors of the NO signaling pathway was able to block the vasorelaxant activity of CAPA (Figure 4), but the concentration-response curve to phenylephrine was shifted to the right in the presence of CAPA (Figure 5), suggesting that high concentrations of CAPA may have α-receptor blocking activity. Because of the relatively low α-adrenergic receptor blocking activity of CAPA, no changes in blood pressure were seen after oral administration of 1, 5, and 10 mg/kg CAPA in the tail cuff plethysmography experiment (data not shown).

With regard to potassium-induced thoracic aorta constriction, CAPA administration relaxed denuded thoracic aorta as shown by an IC_50_ of approximately 75 μM. In endothelium-intact thoracic aorta, CAPA relaxed the thoracic aorta with an IC_50_ of 45 μM. This finding suggests that enhancement of endothelium-dependent NO production may contribute to the relaxant effect of CAPA in endothelium-intact aorta. The relaxant effect of CAPA on potassium-induced constriction in denuded aorta may be mediated by direct inhibition of L-type Ca^2+^ channels in smooth muscle. However, this remains to be investigated.

This study showed that CAPA has potent plasma glucose lowering and coronary artery dilation effects without influencing heart rate and blood pressure. We found decreased plasma glucose levels and increased coronary dilation in CAPA-treated rats; whether CAPA-enhanced vasodilation occurs in blood vessel flow through other tissues remains unknown.

STZ-induced diabetic rats showed decreased autonomic activity and coronary artery perfusion [53]. Our study strongly suggests that CAPA is a powerful candidate for treating vascular disease in diabetic patients.

## Conclusions

CAPA, a compound derived from CAPE, the active component in propolis, shows more structural stability than CAPE and lowers plasma glucose levels and exerts coronary artery dilation effects in normal and diabetic rats. CAPA also ameliorates vascular dysfunction in diabetic rats, suggesting that it could be a good candidate for the treatment of vascular complications in diabetic patients. Since the major complication in diabetes is cardiovascular dysfunction, further studies on cardiac functions, such as on the contractile response in cardiomyocytes, in vivo cardiac hemodynamic function, and underlying molecular mechanisms are required.

## Competing interests

The authors declare that they have no competing interests.

## Authors’ contributions

Participated in research design: M-JS, and W-PC; Conducted experiments: diabetic animal induction, hypoglycemic activity measurement, insulin level measurement and intravenous glucose tolerance test: Y-JH and T-CC; vasomotor activity measurement: Y-JH and C-CCC; retrograde-perfused heart: Y-JH and A-SL; K_ATP_ channel activity in MIN6 cell: W-PC; synthesis, purification of new compound: H-LC and Y-HK; performed data analysis: Y-JH, W-PC and M-JS; Wrote or contributed to the writing of the manuscript: Y-JH, W-PC, A-SL, and M-JS. All authors read and approved the final manuscript.
